# 310. Cryptococcal Infection Following COVID-19 infection in Solid Organ Transplant Recipients: A Case Series

**DOI:** 10.1093/ofid/ofab466.512

**Published:** 2021-12-04

**Authors:** Jeremey Walker, Peter Pappas, Lauren Nicholas Herrera, Cameron White, Anoma Nellore, Todd P McCarty

**Affiliations:** 1 University of Alabama in Birmingham, Birmingham, AL; 2 University of Alabama at Birmingham, Birmingham, Alabama; 3 UAB, Birmingham, Alabama; 4 University of Alabama at Birmingham; Birmingham VA Medical Center, Birmingham, Alabama

## Abstract

**Background:**

Fungal infections have been identified with or following SARS-CoV-2 infection, most commonly COVID associated pulmonary aspergillosis. *Cryptococcus* species are ubiquitous in the environment and the third most common invasive fungal infection following Solid Organ Transplant (SOT). We describe four cases of concurrent or subsequent cryptococcal infection within 90 days following COVID-19 infection.

**Methods:**

We conducted a retrospective study of patients presenting with proven cryptococcosis either concurrently or within 90 days following COVID-19 diagnosis. Cases were identified March 2020 through May 2021. All were seen at the University of Alabama in Birmingham, a regional referral and comprehensive transplant center. Exemption for this review was approved by our IRB.

**Results:**

Four cases were identified, all were SOT recipients. Case details are provided in Table 1. No patients required ICU level care at any point. COVID-19 treatment included 10 days of increased steroids for 3 patients, remdesivir for 2, and 1 received no treatment for COVID-19. In contrast to the typical time-course for cryptococcal infection post-SOT (median time approx. 500 days post-transplant), three patients were greater than 2 years post-transplant and were without rejection or recent changes in immunosuppression. Patient 1 was less than 6 months post liver-kidney transplant and was diagnosed at time of admission with concurrent COVID-19 and cryptococcal pneumonia. Infection was disseminated in the other 3 cases including positive blood cultures in 2 patients and cryptococcal meningitis (CM) in 2 patients. CM cases presented later following COVID-19 and had the longest delay between symptom onset (headache, neurologic symptoms) and CM diagnosis. One patient had CM 8 years prior, but had done extremely well off fluconazole for over 6 years prior to this recurrence. All patients are doing well at most recent follow-up evaluations.

Table 1. Summary of Cases

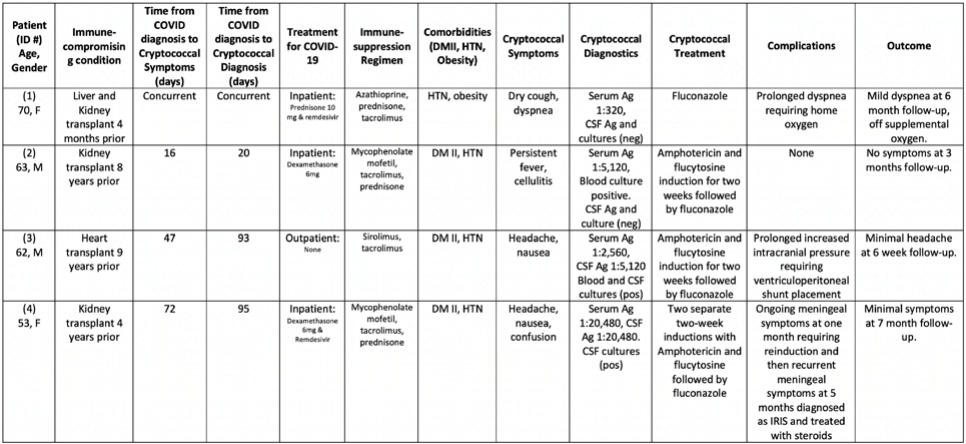

**Conclusion:**

We describe the first case series with a temporal association between SARS-CoV-2 infection and cryptococcosis. All cases were immunocompromised due to SOT. Some symptoms were attributed to post-COVID syndrome leading to significant delays in diagnosis for those patietns, highlighting the importance of considering this association for at-risk patients.

**Disclosures:**

**Todd P. McCarty, MD**, **Cidara** (Grant/Research Support)**GenMark** (Grant/Research Support, Other Financial or Material Support, Honoraria for Research Presentation)**T2 Biosystems** (Consultant)

